# Diagnosis and Treatment of Renal ANCA Vasculitis: A Summary of the Consensus Document of the Catalan Group for the Study of Glomerular Diseases (GLOMCAT)

**DOI:** 10.3390/jcm13226793

**Published:** 2024-11-12

**Authors:** Juliana Bordignon Draibe, Helena Marco, Meritxell Ibernon, Irene Agraz, Carola Arcal, Xoana Barros, Victoria Cabrera, Iara Da Silva, Montserrat Díaz, Xavier Fulladosa, Elena Guillén, Patricia Lescano, Laura Martínez Valenzuela, Eva Márquez, Nadia Martín, Ana Merino, Maru Navarro, Eva Rodríguez, Mª José Soler, Joan Torras, Luís F. Quintana, on behalf of the Catalan Society of Nephrology

**Affiliations:** 1 Nephrology Department, Hospital Universitari de Bellvitge, L’Hospitalet de Llobregat, 08907 Barcelona, Spain; jbordignon@bellvitgehospital.cat (J.B.D.); xfulladosa@bellvitgehospital.cat (X.F.); lmartinezv@bellvitgehospital.cat (L.M.V.); jtorras@bellvitgehospital.cat (J.T.); 2 Nephrology Department, Fundació Puigvert, 08025 Barcelona, Spain; hmarco@fundacio-puigvert.es (H.M.); xbarros@fundacio-puigvert.es (X.B.); mmdiaz@fundacio-puigvert.es (M.D.); 3 Nephrology Department, Hospital Moisès Broggi, Sant Joan Despí, 08970 Barcelona, Spain; minavarrod@csi.cat; 4 Nephrology Department, Hospital Vall d’Hebron, 08035 Barcelona, Spain; irene.agraz@vallhebron.cat (I.A.); m.soler@vallhebron.cat (M.J.S.); 5 Nephrology Department, Hospital d’Igualada, 08700 Igualada, Spain; carcal@csa.cat; 6 Nephrology Department, Hospital de Palamós, 17230 Palamós, Spain; mcabrera@ssibe.cat (V.C.); plescano.garcia17@gmail.com (P.L.); 7 Nephrology Department, Hospital Germans Trías i Pujol, 08916 Badalona, Spain; iarakarlla@hotmail.com; 8 Nephrology Department, Hospital Clínic de Barcelona, 08036 Barcelona, Spain; eguillen@clinic.cat (E.G.); lfquinta@clinic.cat (L.F.Q.); 9 Nephrology Department, Hospital del Mar, 08003 Barcelona, Spain; eva.marquez.mosquera@psmar.cat (E.M.); erodriguezg@psmar.cat (E.R.); 10 Nephrology Department, Hospital Josep Trueta, 17007 Girona, Spain; nadiamartin.girona.ics@gencat.cat (N.M.); anamerinoribas@gmail.com (A.M.)

**Keywords:** anti-neutrophil cytoplasmic antibody-associated vasculitis, granulomatosis with polyangiitis, microscopic polyangiitis, eosinophilic granulomatosis with polyangiitis

## Abstract

The document provides a comprehensive overview of the diagnosis, monitoring, and treatment of anti-neutrophil cytoplasmic antibody-associated vasculitis (AAV) with renal involvement, focusing on granulomatosis with polyangiitis (GPA) and microscopic polyangiitis (MPA). It outlines the definitions, clinical presentation, histopathological classification, monitoring strategies, induction and maintenance treatments, as well as special considerations for relapsing, refractory, and frail patients with renal AAV. The document was prepared by the Catalan Group for the Study of Glomerular Diseases (GLOMCAT), which comprises nephrologists with extensive experience in the diagnosis and treatment of AAV patients. Several virtual and face-to-face meetings were held for coordination, section assignments, and content discussion. An exhaustive and systematic search of the literature was carried out, which included, among others, the following databases: PubMed, EMBASE, Cochrane Library, Google Scholar, and ClinicalTrials.gov, as well as the abstract books of national and international congresses. Overall, the document provides a comprehensive guide for clinicians managing patients with renal AAV, offering evidence-based recommendations for diagnosis, monitoring, and treatment across various clinical scenarios.

## 1. Introduction

Anti-neutrophil cytoplasmic antibody-associated vasculitis (AAV) is a group of systemic diseases characterized by inflammation and destruction of small blood vessels in the presence of circulating anti-neutrophil cytoplasmic antibodies (ANCA). There are three clinical entities that fall under the AAV classification: granulomatosis with polyangiitis (GPA), microscopic polyangiitis (MPA), and eosinophilic granulomatosis with polyangiitis (EGPA).

In AAV, the kidney is involved in 16–60% of patients, and this is characterized by rapidly progressing kidney failure due to necrotizing glomerulonephritis with extracapilary proliferation and minimal or no immune-complex deposition on vessel walls (pauci-immune; Supplementary Figure S1) [1].

Recently, several randomized clinical trials have changed our clinical practice regarding the treatment of renal AAV. The combination of different therapies and the emergence of new drugs that block specific pathways allows for the reduction of corticosteroid dosage and leads to an increase in the number of patients achieving complete remission of the disease [2,3].

The primary aim of this document is to compile, assess, and condense the latest information on renal AAV into a user-friendly and practical format, facilitating enhanced management of AAV patients. The condensed version we present here focuses on crucial aspects of the consensus document, concentrating on the diagnosis, definitions, and treatment approaches of renal AAV, along with addressing special situations. Recognizing the wealth of valuable documents and guidelines on AAV management, such as the 2022 EULAR/ERA–EDTA recommendations [4], our consensus relies on the management of renal involvement in AAV focused on GPA and MPA, adding strategies in easily interpretable algorithms and tables, streamlining their translation to clinical practice.

The document was prepared by the Catalan Group for the Study of Glomerular Diseases (GLOMCAT), which is composed of nephrologists with extensive experience in the diagnosis and treatment of AAV patients. Several virtual and face-to-face meetings were held for coordination, section assignments, and content discussion. An exhaustive and systematic search of the literature was carried out, which included, among others, the following databases: PubMed, EMBASE, Cochrane Library, Google Scholar, and ClinicalTrials.gov, as well as the abstract books of national and international congresses.

Definitions of the AAV activity states differ among clinical trials. For the purposes of this protocol, we propose the following definitions based on the recommendations of EULAR (European Alliance of Associations for Rheumatology) for AAV clinical trials and KDIGO guidelines Table 1 [4].

“Evidence was graded according to the Levels of Evidence and Grades of Recommendation by the Centre for Evidence-Based Medicine (Oxford University). Levels of evidence range from systematic reviews and randomized controlled trials (highest quality) to expert opinion (lowest quality). Grades of recommendation, based on the strength of evidence, range from A (a strong recommendation based on consistent evidence) to D (a weak recommendation due to limited or conflicting evidence; see Supplementary Table S1).

## 2. Clinical Presentation and Clinical/Histopathological Diagnosis

**Figure 1:**
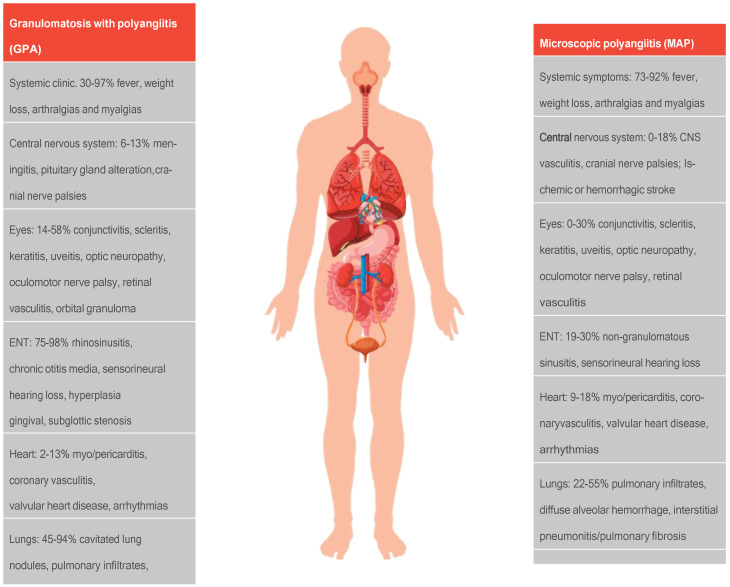
Clinical manifestations of ANCA-associated renal vasculitis. CNS: central nervous system; CVA: cerebrovascular accident. (Figure adapted from Diagnosing and treating ANCA-associated vasculitis: an updated review for clinical practice. *Rheumatology* **2022**, 1–17. [7]).

Kidney involvement in ANCA-associated vasculitis is frequent and the most common predictor of mortality. Patients with estimated glomerular filtration rate (eGFR) <50 mL/min have a 50% risk of death or kidney failure within 5 years of diagnosis. The most common presentation is rapidly progressive glomerulonephritis with altered kidney function associated with non-nephrotic proteinuria (between 1 and 3 g/day), microhematuria, and hypertension [6]. Other clinical presentations are detailed in Figure 1 [7].

In 2010, the ACR (American College of Rheumatology) and EULAR began the biggest multinational observational study of vasculitis in history: DCVAS (Diagnostic and Classification Criteria in Vasculitis) to develop classification and diagnostic criteria for GPA, MPA, and EGPA as well as polyarteritis nodosa, giant cell arteritis and Takayasu arteritis [8,9,10]. We recommended this classification for MPA and GPA diagnosis (Table 2 and Table 3).

Kidney biopsy not only confirms the diagnosis of AAV but also provides prognostic insights into renal outcomes. Additionally, it plays a crucial role in managing relapses or refractory cases by assessing the presence of chronic lesions that may not be responsive to immunosuppressive treatment.

**Figure 2:**
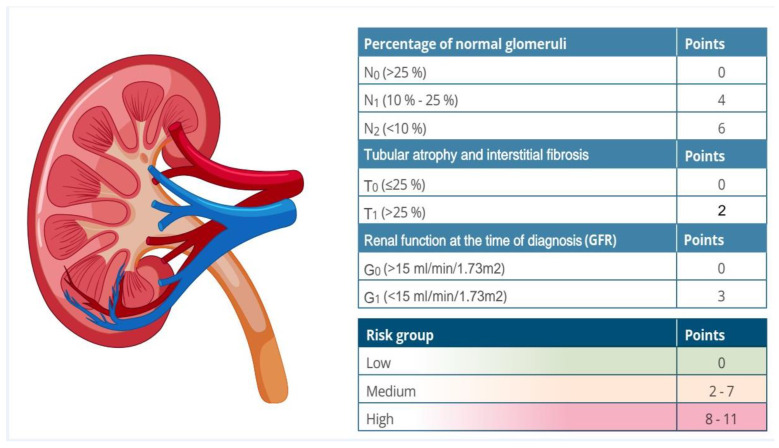
Renal Risk Score.

**Figure 3:**
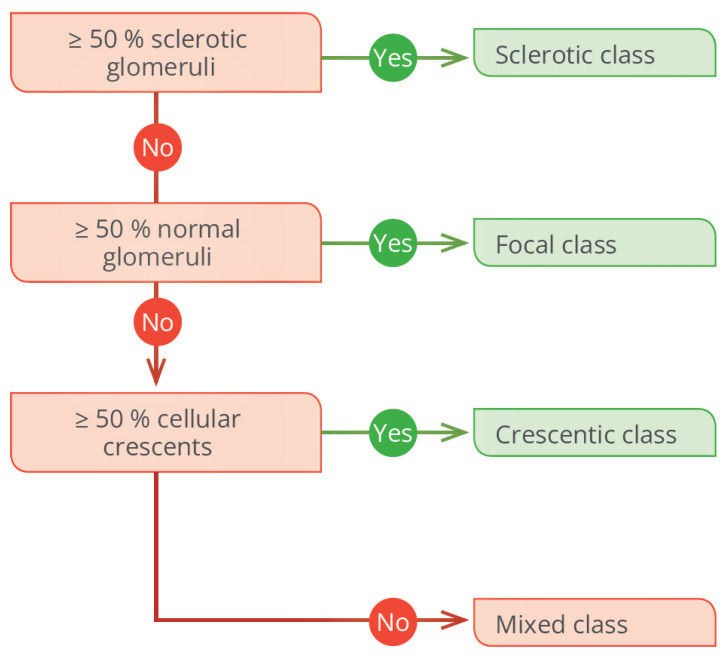
Berden’s histopathologic classification in AAV.

Kidney biopsy can predict the long-term risk of advanced chronic kidney disease (ACKD). Some authors have developed histopathological indices with prognostic value. In the Renal Risk Score developed by Brix et al., a higher percentage of normal glomeruli (>25%) was associated with favorable renal results (Figure 2) [11]. Another widely known histopathological classification is from Berden et al. (Figure 3) [12]. Validation studies have shown that the focal subgroup (≥50% normal glomeruli) is associated with a favorable prognosis, while the sclerotic subgroup (≥50% glomeruli with sclerosis) is associated with an unfavorable prognosis. Nevertheless, discrepancies have been found regarding the mixed and crescentic subgroups (≥50% glomerular crescents) [13,14].

## 3. Monitoring Renal AAV After Diagnosis

We don’t currently have a specific biomarker capable of determining the renal activity of vasculitis (Level of Evidence B) during the progression of the disease or that can predict a relapse, persistent case, or even response to treatment. This chapter looks at the markers normally used in daily clinical practice.

The markers utilized to evaluate the presence of renal involvement include serum creatinine, estimated glomerular filtration rate, hematuria, and proteinuria. When acute glomerulonephritis is histologically confirmed, the presence of hematuria is indicative of active lesions. The significance of persistent hematuria once the remission stage is reached is unknown. Unfortunately, hematuria and proteinuria persist in remission in up to 50% of patients, thus complicating the use of urinalysis to evaluate renal AAV activity. In these cases, kidney function, extrarenal manifestations, and inflammatory markers may be useful. A retrospective study did not find differences in the risk of developing chronic kidney disease in patients with persistent hematuria compared to those without persistent hematuria, although a higher proportion of patients with hematuria developed a renal flare during follow-up. Similarly, persistent proteinuria can indicate AAV activity or chronic damage, thus not being a useful marker itself. Conversely, the reappearance of hematuria during remission has been associated with disease activity.

The diagnostic utility of serum ANCA measurements in an appropriate clinical context is indisputable, although the value of these serial tests in monitoring the disease and their ability to predict vasculitis outbreaks is highly controversial. A prior meta-analysis showed that serial ANCA measurements are not useful in predicting AAV outbreaks, although Kemna et al. showed the ability of the serial titration of ANCA to predict AAV flares specifically involving the kidney [15]. Therefore, the kinetics of ANCA titers during follow-up should not be utilized in decision-making. However, patients with persistently positive ANCA or rising titer should be closely monitored. Negativisation of ANCA after induction treatment, on the contrary, is associated with a low risk of relapse [16,17].

**Figure 4:**
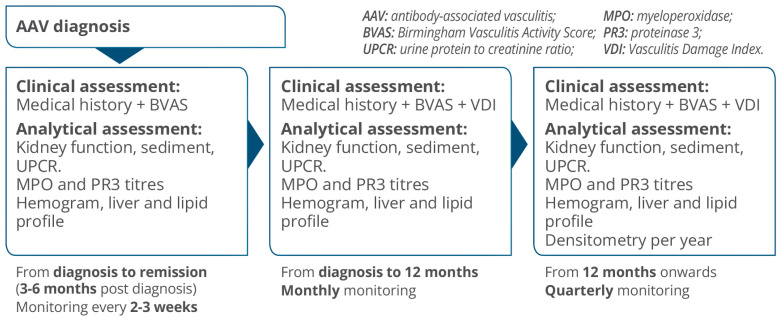
AAV Monitoring algorithm.

Based on this information, our group has developed a practical algorithm for monitoring and tracking patients with renal AAV (Figure 4).

## 4. Induction and Maintenance Treatment

### 4.1. Induction Treatment

Corticosteroids are recommended in combination with cyclophosphamide or rituximab as an initial treatment AAV with kidney involvement (Level of Evidence B). In patients with severe kidney involvement, plasma exchange and avacopan should be considered (Level of Evidence B). In that case, there is currently not enough evidence to recommend treatment with rituximab alone (Level of Evidence B) [18].

Steroid regime: Starting treatment with oral prednisone (1 mg/kg) with a tapering schedule, as indicated in Table 4, is recommended (Level of Evidence 1B). It has been shown that a reduced dose of glucocorticoids is not inferior to the standard regimen and reduces the risk of adverse events associated with therapy [19]. 

There is insufficient evidence to endorse the regular utilization of methylprednisolone pulse therapy in conjunction with oral prednisone induction therapy. Further research is necessary to explore and elucidate this subject (Level of Evidence C) [20].

Based on that information, consider administering pulses of 0.5–1 g of methylprednisolone (administered for 3 consecutive days), with a maximum cumulative dose between 1–3 g prior to initiation of the oral regimen only in cases of severe renal failure or pulmonary hemorrhage (Level of Evidence UG).

Cyclophosphamide: The efficacy of cyclophosphamide, administered with prednisone, to treat AAV has been established in various clinical trials. Oral and intravenous administration of cyclophosphamide can be used. In 2009, the CYCLOPS study showed no differences in remission induction between intravenous pulse and oral administration, achieving a reduced-dose regimen with pulse treatment and less leukopenia, although pulse treatment was associated with a greater risk of a future relapse in the extended trial but not associated with a decrease in kidney or patient survival [21,22].

Based on that information, we recommend treatment with intravenous pulses as the first option, as it reduces the cumulative dose administered. The recommended intravenous dose consists of 3 doses of cyclophosphamide every 2 weeks, followed by three doses every 3 weeks. Intravenous pulses are recommended, with an initial dose of 15 mg/kg/pulse, not to exceed 1.2 g per pulse. The tapering regimen is applied based on age and kidney function (Level of Evidence UG; Table 5).

Rituximab: The efficacy of rituximab, administered with prednisone, to treat ANCA-associated vasculitis with non-severe renal involvement (creatinine ≤354 µmol/L, ≤4 mg/dL, FG ≥ 15 mL/min) has been demonstrated in the RAVE trial, that analyzed non-inferiority compared to standard treatment with cyclophosphamide. Treatment with rituximab is also recommended in cases of relapsing vasculitis (Level of Evidence 1B) [18].

Treatment with rituximab is recommended as the first choice for non-severe vasculitis whenever possible (Level of Evidence B). Two regimens can be used: rituximab 1 g at week 1 and week 2 or 375 mg/m^2^/week for 4 weeks (Table 6).

Cyclophosphamide+ Rituximab: Combined treatment with intravenous cyclophosphamide and rituximab, with prednisone, is recommended in patients with severe kidney involvement (creatinine >354 µmol/L, >4 mg/dL, FG < 15 mL/min; Level of Evidence B).

This option allows for a lower cumulative dose of cyclophosphamide with the same efficacy and without major side effects. There are two possible regimens:

According to the RITUXVAS trial, rituximab 375 mg/m^2^/week for 4 weeks with two doses of cyclophosphamide based on age and kidney function (Level of Evidence 1B) [23].

According to the CycLowVas prospective cohort trial: rituximab 1 g at week 0 and week 2 with six doses of cyclophosphamide every 2 weeks (the first two doses at 10 mg/kg not to exceed 750 mg and the following four doses not to exceed 500 mg; Level of Evidence 2B) [3].

Mycophenolate Mofetil: Based on MYCYC trial, in the subgroup of patients with MPO-positive vasculitis with limited kidney involvement, mycophenolate can be considered as an option (Level of Evidence 1B). The recommended doses are 2 g per day, or the maximum dose tolerated between 1–2 g/day for 3–6 months until remission. Patients with rapidly progressive deterioration of kidney function and/or GFR < 15 mL/min should be excluded (Level of Evidence UG) [24].

Plasma Exchange: Treatment with plasma exchange is recommended in the following cases (Level of Evidence B) [25]:

1. Patients with serum creatinine at onset >300 µmol/L or who require renal replacement therapy.
2. For patients with positive ANCA and glomerular basement membrane antibodies (anti-GBM), it is also indicated to associate plasma exchange (PE) [2], as these patients behave similarly to patients with anti-GMB disease.

Treatment with plasma exchange can be considered (Level of Evidence D):

1. Patients with diffuse alveolar hemorrhage associated with hypoxemia, if they do not respond to steroid treatment

Plasma exchange regimen (PE) (Level of Evidence 1B): The PE regimen from the PEXIVAS trial is recommended [19]: seven treatments over no more than 14 days, with an exchange volume of 60 mL/kg of body weight with albumin replacement. Intravenous immunoglobulin (IVIg) was not used after PE. In patients with active bleeding (alveolar hemorrhage) or high risk of bleeding, administer PE daily until bleeding ceases and use fresh frozen plasma instead of albumin as a replacement solution. Patients with positive ANCA and anti-GBM: PE daily for 14 days or until anti-GBM titers are negative.

Avacopan: Avacopan is an orally administered small-molecule C5a receptor antagonist that selectively blocks the effects of C5a through the C5a receptor (C5aR), including blocking neutrophil chemoattraction and activation [2].

In the phase 3 Advocate trial, patients were assigned to receive oral avacopan or oral prednisone on a tapering schedule associated with cyclophosphamide (followed by azathioprine) or rituximab as an induction treatment. In this trial, avacopan was noninferior to prednisone taper in achieving remission at week 26 and was superior to prednisone taper in sustaining remission at week 52. More than that, a post-hoc analysis confirmed that in patients with a baseline eGFR ≤20 mL/min per 1.73 m^2^, eGFR improved more in the avacopan group than in the prednisone group [2].

Based on these data, we recommended avacopan in combination with a regimen of rituximab or cyclophosphamide for the treatment of adult patients with severe and active GPA or MPA, mostly for specific subgroups as patients at risk of steroid toxicity and individuals with active glomerulonephritis and rapidly declining kidney function (with baseline eGFR ≤20 mL/min) (Level of Evidence 1B).

Precautions for the use of Avacopan and criteria for current non-financing by the Ministry of Health in Spain:

1. Patients under 18 years of age.
2. Different forms of vasculitis other than GPA or MPA
3. Acute or chronic infection with hepatitis B virus (HBV), hepatitis C virus (HCV), human immunodeficiency virus (HIV), or tuberculosis.
4. Evidence of severe liver disease (Child-Pugh class C).
5. Alveolar hemorrhage requiring invasive pulmonary ventilation.
6. Pregnancy and women of childbearing age who are not using contraceptive methods.
7. eGFR <15 mL/min/1.73 m^2^, need for dialysis or plasma exchanges.

The daily dose is 30 mg (3 capsules) every 12 h.

Related to tapering the dosage of prednisone, we recommend decreasing the prednisone regimen in combination with avacopan until complete withdrawal in 4 weeks (Level of evidence 1B) [2]. Do not withdraw prednisone if the patient has an indication for treatment with glucocorticoids (GC) for a reason other than AAV (Level of evidence D).

### 4.2. Maintenance Treatment

The best maintenance strategy for AAV patients in remission has not been clearly established. Administering cyclophosphamide for more than 3 months is not generally recommended due to possible severe toxicity, as other strategies are available. Treatment with rituximab is recommended or, as a second option, azathioprine and glucocorticoids in low doses after remission induction (Level of Evidence 1B).

Rituximab: Rituximab in the maintenance of remission is associated with a lower rate of relapse after induction with cyclophosphamide (MAINRITSAN trial) and also when induction has been carried out with rituximab (RITAZAREM trial), both compared with maintenance treatment with azathioprine [26,27]. Different doses of rituximab have been used to maintain remission in these clinical trials, and no comparative trials have been performed to assess the optimal dose. In the RITAZAREM study, the rituximab regimen applied was five doses of 1 g every 4 months. In the MAINRITSAN trial, after induction with cyclophosphamide, rituximab was started at 6 months (two doses of 500 mg, days 1 and 14), and subsequently, three more doses every 6 months were administered. In a second study, the same group compared this fixed regimen of rituximab with a regimen based on ANCA levels and/or the reappearance of B cells before the next infusion. Although dosing based on the reappearance of B cells and ANCA requires fewer infusions of rituximab, there is a non-statistically significant increase in the appearance of relapses [28].

Therefore, the initial recommendation from this working group is to administer rituximab in a fixed regimen every 6 months to maintain remission. In case of relapse, consider an additional dose of rituximab if at least 4 months have passed since the last administration (Level of Evidence 1B).

Testing for HVB, HCV, and interferon-gamma release assays (IGRAs) prior to the initiation of the treatment is advised. To evaluate the patient’s immunological state during treatment, monitoring B cells (CD19) immunoglobulins (IgG) is recommended. If IgG < 300 mg/L, treatment with immunoglobulins may be necessary (Level of Evidence 1B).

Azathioprine: If rituximab is not available or appears inappropriate (the patient is allergic to rituximab or presents severe hypogammaglobulinemia), azathioprine is recommended. 

Azathioprine has shown similar remission maintenance levels at 18 months as Cyclophosphamide maintenance treatment, although it has shown an increase in recurrent cases from 18 months compared to maintenance treatment with RTX [26,29]. Azathioprine is also superior to mycophenolate mofetil for remission maintenance [30].

The recommended dose is 2 mg/kg/day for the first 12 months; after the first 12 months, the dose should be reduced by 25 mg every 3 months. The total daily dose should be divided into two doses, with a maximum of 200 mg/day. Doses should be adjusted by age. In patients >60 years old, reduce the dose by 25% (1.5 mg/kg/day). In patients >75 years old, reduce the dose by 50% (1 mg/kg/day).

Mycophenolate mofetil: Among patients with vasculitis, mycophenolate mofetil was less effective than azathioprine in maintaining remission of the disease in comparative studies, although both treatments had similar adverse event rates [30]. Mycophenolate mofetil can be used in cases of contraindications, intolerance, or allergy to azathioprine or rituximab. The recommended doses are 1000 mg (oral) of mycophenolate mofetil twice a day or Myfortic^®^ 720 mg twice a day. 

Steroid Maintenance: There is not sufficient evidence to support a specific dose of glucocorticoid treatment during the maintenance phase, and it must be determined based on the clinical circumstances of each patient. Many patients require some treatment with glucocorticoids, generally at low doses, to keep nonrenal symptoms under control. The minimum effective dose should be prescribed to minimize glucocorticoid toxicity, and from week 50, proceed based on the experience of the center.

Duration of maintenance treatment after remission: There is no proven evidence for the minimum length of maintenance treatment; a longer maintenance period reduces relapse rates, but it is also associated with more adverse events.

Results from the Remain study showed that maintaining treatment with azathioprine/prednisone beyond 24 months up to 4 years after diagnosis is associated with a decreased risk of relapse and better kidney survival. The authors did not find differences in the relapse rate according to ANCA type or titer within 12 months since diagnosis [31]. On the other hand, the MAINRITSAN-3 study demonstrated a superior risk of relapse in patients treated for 36 months compared to 18 months with rituximab [32].

So, in this working group, the recommended maintenance treatment duration is 24–48 months (Level of Evidence 1B). Several factors related to the increased risk of relapse have been taken into account in this decision: prior history of relapse, ANCA state and characteristics (with PR3-ANCA positive patients with a higher chance of relapse), the persistence of ANCA after induction therapy (associated with a higher risk of relapse), and intensity of induction therapy [10].

There is no evidence regarding whether to maintain treatment once the patient is in end-stage kidney disease requiring renal replacement therapy. Some authors suggest maintaining therapy for up to 3–6 months, depending on the severity of the extrarenal manifestations and the eventual possibility of kidney function recovery, although other authors did not find any benefit in maintaining treatment with CF beyond 4 months [33,34].

The appropriate time for kidney transplantation in patients with ANCA-associated vasculitis is not clear. There is an increased risk of graft failure due to death if transplanted in the first 12 months of remission. Therefore, the recommendation is to wait at least 1 year after clinical remission before a kidney transplant. The presence of ANCA-positive titers does not exclude transplantation. However, the presence of PR3-specific ANCA is associated with a higher risk of relapse after transplantation, and these patients must be monitored more closely. Treatment of ANCA-associated vasculitis outbreaks after transplantation is similar to treatment prior to transplantation, taking into account the characteristics and severity of the outbreak [35] and tailoring the immunosuppressive regimes.

**Figure 5:**
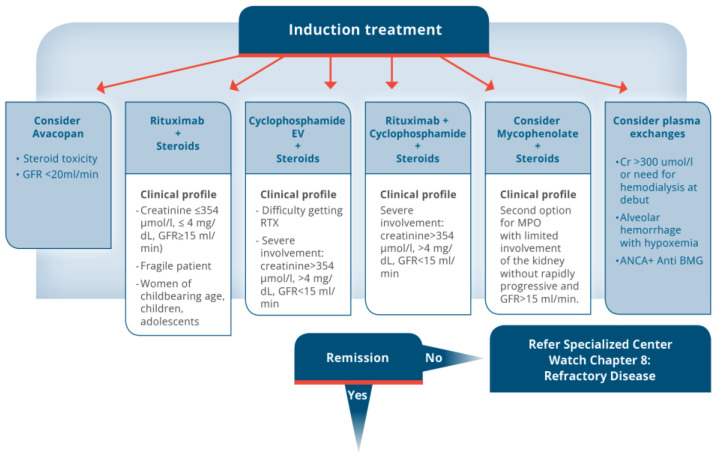

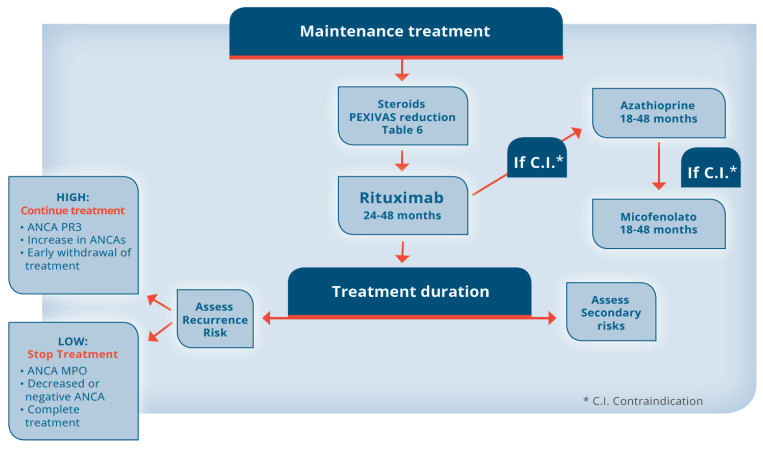
Algorithm Renal ANCA VASCULITIS.

Based on all the information regarding induction and maintenance treatment, our group generated a treatment algorithm (Figure 5).

## 5. Special Situations

### 5.1. Relapsing Disease

The response rate to immunosuppressive treatment in relapses is similar to the response at disease onset. To manage relapses, it is important to take into account whether the patient is taking maintenance immunosuppressive treatment, the severity of the relapse, and the induction therapy received previously, particularly the cumulative dose of cyclophosphamide [10,36].

Rituximab is the first line therapy in relapse patients (Level of Evidence 1B), given evidence from post-hoc analysis of the RAVE randomized controlled trial, in which a higher remission rate was observed in relapsing patients treated with rituximab compared to cyclophosphamide [37]. There is evidence that mycophenolate is useful as induction to remission treatment in vasculitis with minor relapses (Level of Evidence 1B) [24,38]. Relapse treatment with cyclophosphamide has a Level of Evidence 1A according to EULAR/EDTA 2016 guidelines [39]. If the decision is to treat with cyclophosphamide, it must be taken into account that a cumulative dose above 36 g has been associated with various neoplasms in patients with AAV [40]. The clinical practice guidelines of the American College of Rheumatology only recommend relapse treatment with cyclophosphamide if the patient is on maintenance treatment with rituximab and has received a dose recently [41]. 

Our Recommendation is to resume/increase the dose of steroids as the first therapeutic step (Level of evidence UG) associated with rituximab. In cases of severe relapses, contraindications, or recent dosage of rituximab, cyclophosphamide could be an option.

**Figure 6:**
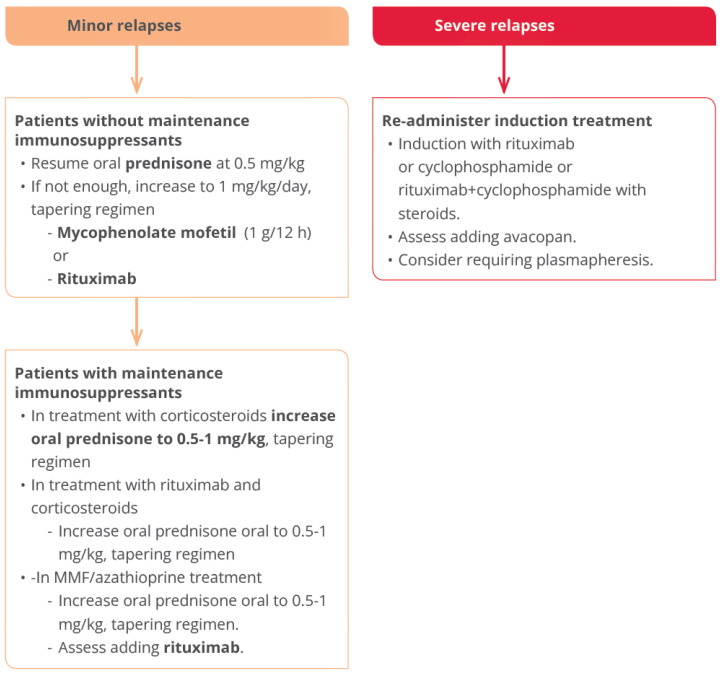
Algorithm to treat renal ANCA vasculitis relapses.

We propose the algorithm above to treat relapse (Figure 6):

### 5.2. Refractory Disease

Refractory disease is defined as the failure to significantly suppress disease activity, indicated by a decrease of at least 50% in the BVAS score at diagnosis 12 weeks after the implementation of the induction to remission [36] First, the cause of refractory disease: drug intolerance, comorbidities, vasculitis triggers (drugs, infection, neoplasms) must be determined, before classifying the clinical situation as refractory [10].

Our recommendation is to initially consider increasing the dose of prednisone and thereafter associate rituximab or cyclophosphamide (switching from the chosen induction treatment). Add plasma exchange if required (Level of evidence C) [10,41].

In cases of corticosteroid dependence, resistance, or toxicity, adding avacopan to remission, induction treatment has been described as useful [42] (Level of evidence UG).

The use of immunoglobulins in patients with persistent activity at a dose of 2 g/kg for short periods has also been proposed (Level of evidence UG) [10,36,43].

### 5.3. Treating Frail Patients with Renal AAV

**Figure 7:**
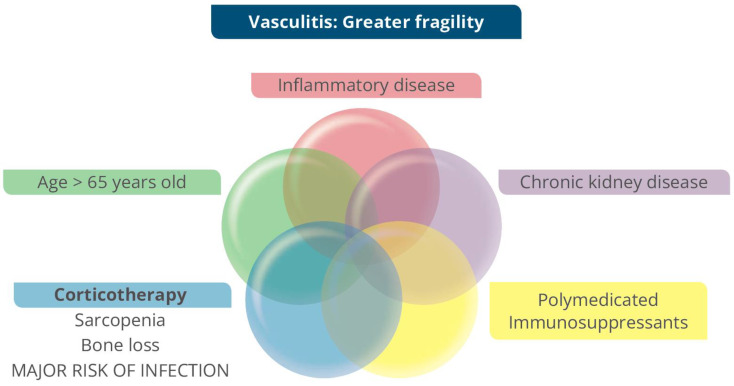
Renal ANCA Vasculitis in frail patients.

Frailty is defined as a syndrome characterized by greater vulnerability that leads to diminished reserves and functional decline, causing reduced resistance to stressors. Frailty leads to an increased risk of adverse health outcomes, including falls, disability, hospitalization, and mortality. Fragility is a syndrome frequently seen in the elderly and those with chronic illnesses, regardless of their age, such as chronic inflammatory disease and chronic kidney disease. So, patients with vasculitis, by definition, are considered fragile patients regardless of their age (Figure 7) [44,45,46,47].

ANCA-associated vasculitis is more prevalent among the elderly, with a peak incidence between 65 and 74 years old. In elderly patients, AAV diagnosis tends to take longer, as symptoms of the disease, like asthenia and toxic shock syndrome, tend to be masked and attributed to age. Moreover, AAV in the elderly occurs in a context of accompanying comorbidities (for example, heart disease, diabetes, or cancer), which could explain the higher mortality rate for AAV in this age group, especially during the first year of treatment and be associated with the presence of kidney involvement. The main cause of mortality in this age group is complications from AAV treatment, mainly infectious complications, making it necessary to minimize treatment and accentuate infectious prophylaxis and specific vaccination programs [48,49].

There are no randomized clinical trials comparing the various immunosuppressive treatments in the fragile population. Moreover, these patients are under-represented in existing AAV-controlled clinical trials. McGovern et al. conducted a prospective observational study with a large cohort—both in the number of patients recruited and time monitored—on the use of corticosteroids in elderly patients [50]. According to the results of this study, low doses of corticosteroids (avoiding intravenous methylprednisolone) are effective in treating patients over 75 years with good long-term clinical results. In this group, the use of IV methylprednisolone was associated with a greater risk of infection. Furthermore, Watanabe-Imai et al. [51] assessed treatment-related damage in patients with elderly onset AAV. This study showed that glucocorticoid doses >0.8 mg/kg/day were a risk factor for serious infection in elderly patients. 

Additionally to steroids, rituximab can have advantages in elderly patients, avoiding the toxicity of cyclophosphamide, but data are limited for this population. A recently published study was conducted on 93 patients over 75 years old with AAV treated with rituximab as remission induction and maintenance treatment. Rituximab therapy was associated with remission in 86% of cases and with remission maintenance in the majority of patients [52]. The KDIGO guidelines recommend avoiding cyclophosphamide in this group of patients. If there is no alternative, they recommend decreasing the maximum dose from 15 mg/kg to 12.5 mg/kg in patients over 60 and to 10 mg/kg in patients over 70 [10]. In severe cases, the combination of rituximab and cyclophosphamide in low doses yields quick control over the disease and prolonged remission and makes it possible to minimize the use of glucocorticoids [10].

Based on that data n, our group recommends avoiding intravenous methylprednisolone and only considering this option in severe cases, when three pulses of 250 mg EV with a fast tapering regimen (Table 4) of glucocorticoids is recommended (level of evidence B). As an alternative to avoid steroids in this group of patients, the use of avacopan could be considered (level of evidence D). In association with steroids, Rituximab should be added to avoid cyclophosphamide toxicity (level of evidence B). In severe cases, the combination of rituximab and a low dosage of cyclophosphamide could be an option (level of evidence D).

## Figures and Tables

**Table 1:** Disease activity states in AAV.

**Disease activity**	Signs and/or symptoms of active AAV in any organ. One of the methods for assessing disease activity is the *Birmingham Vasculitis Activity Score* (Birmingham Vasculitis activity score: BVAS) [5]
**Remission**	Absence of clinical manifestations of active vasculitis or glomerulonephritis. Specifically for glomerulonephritis, it is defined as the improvement or stability of glomerular filtration. While proteinuria and haematuria are present when the disease is active and can be resolved completely, their persistence does not necessarily imply disease activity.
**Relapse**	Recurrence of active AAV after a period of full or partial remission. An increase or reappearance of proteinuria or haematuria can indicate kidney relapse. Relapses can be broken down into two types: **Severe relapses:** Recurrences that are life-threatening or may result in significant organ damage or dysfunction. For example, diffuse alveolar hemorrhage, glomerulonephritis, etc. **Minor relapses:** These are not life-threatening or do not lead to significant impairment of organ function
**Resistance to treatment or refractory disease**	Persistence or reappearance of kidney and/or systemic manifestations of ANCA-associated vasculitis with the same intensity of treatment as at the onset of the disease. Infections, side effects of treatment, comorbidities, and organ damage must be ruled out.

**Table 2:** Granulomatosis with polyangiitis classification criteria (ACR/EULAR 2022).

**In applying these criteria, the following must be considered:** **The classification criteria can be used to classify the disease as granulomatosis with polyangiitis if the patient has been diagnosed with small- or medium-vessel vasculitis** **The patient is excluded from alternative diagnoses of diseases that mimic vasculitis**	
**Clinical criteria**	**Points**
Nasal involvement: bloody nasal discharge, ulcers, nasal crusting, sino-nasal congestion, nasal respiration disorder, nasal septum defect or perforation	**+3**
Cartilaginous involvement: inflamed ear or nose cartilage, hoarse voice/stridor, endobronchial involvement, or saddle nose	**+2**
Conductive or sensorineural hearing loss	**+1**
**Laboratory, morphology, or instrumental criteria**	
cANCA or PR3-antibody positive	**+5**
Nodules, masses, or cavitation on chest imaging	**+2**
Granuloma, extravascular granulomatous inflammation, or giant cells on biopsy	**+2**
Inflammation, consolidation, or effusion of the nasal/paranasal sinuses on imaging	**+1**
Pauci-immune glomerulonephritis on kidney biopsy	**+1**
pANCA or MPO-ANCA positive	**−1**
Eosinophil count ≥1 × 10^9^/L	**−4**
Add up the patient’s points for all the criteria. A total score ≥ 5 is needed for the classification of granulomatosis with polyangiitis.	

Note. Here and later in Table 2 and Table 3: cANCA: cytoplasmic ANCA; PR3-ANCA: proteinase-3 ANCA; pANCA: perinuclear ANCA; MPO: myeloperoxidase; MPO-ANCA: myeloperoxidase ANCA.

**Table 3:** Microscopic polyangiitis classification criteria (ACR/EULAR 2022).

**In applying these criteria, the following must be considered:** **The classification criteria can be used to classify the disease as microscopic polyangiitis if the patient has been diagnosed with small- or medium-vessel vasculitis** **The patient is excluded from alternative diagnoses of diseases that mimic vasculitis**	
**Clinical criteria**	***Points***
Nasal involvement: bloody nasal discharge, ulcers, nasal crusting, sino-nasal congestion, nasal respiration disorder, nasal septum defect or perforation	**−3**
**Laboratory, morphology, or instrumental criteria**	
pANCA or MPO-ANCA positive	**+6**
Pulmonary fibrosis or interstitial lung diseases on chest imaging	**+3**
Pauci-immune glomerulonephritis on kidney biopsy	**+3**
cANCA or PR3-antibody positive	**–1**
Eosinophil count ≥1 × 109/L	**–4**
Add up the patient’s points for all the criteria. A total score ≥5 is needed for the classification of microscopic polyangiitis.	

**Table 4:** Tapering regimen for glucocorticoids in the PEXIVAS trial.

Tapering Regimen for Glucocorticoids in the PEXIVAS							
Week	<50 kg	50–75 kg	>75 kg	Week	<50 kg	50–75 kg	>75 kg
**1**	50	60	75	**13–14**	6	7.5	10
**2**	25	30	40	**15–16**	5	5	7.5
**3–4**	20	25	30	**17–18**	5	5	7.5
**5–6**	15	20	25	**19–20**	5	5	5
**7–8**	12.5	15	20	**21–22**	5	5	5
**9–10**	10	12.5	15	**23–52**	5	5	5
**11–12**	7.5	10	12.5	**>52**	Local practice		

**Table 5:** Cyclophosphamide dose based on age and kidney function.

Age (Years)	MDRD–CKD-EPI >30 mL/min	MDRD–CKD-EPI <30 mL/min
**<60**	Full dose	85%
**60–70**	85%	65%
**<70**	65%	50%

**Table 6:** Factors to consider when choosing between rituximab and cyclophosphamide.

Preference for Rituximab	Preference for Cyclophosphamide
Children and adolescents	Difficulties getting rituximab
Men and women with an interest in reproducing	Vasculitis with severe kidney involvement and/or alveolar hemorrhage Consider combined treatment
Fragile patients	
Rapid reduction of prednisone	
Recurrent vasculitis	
PR3 positive vasculitis	

## Data Availability

Not applicable.

## Supplementary Materials

The following supporting information can be downloaded at: https://www.mdpi.com/article/10.3390/jcm13226793/s1, Figure S1: Necrotizing glomerulonephritis with extracapilary proliferation in a patient with ANCA Associted vasculitis, Table S1: Evidence was graded according to the *Levels of Evidence and Grades of Recommendation* by the Centre for Evidence-Based Medicine (Oxford University)
