# Supplementary material for: Diagnosis and Treatment of Renal ANCA Vasculitis: A Summary of the Consensus Document of the Catalan Group for the Study of Glomerular Diseases (GLOMCAT)
Source: J Clin Med. 2024 Nov 12;13(22):6793. doi: 10.3390/jcm13226793 (PMC11594726; doi:10.3390/jcm13226793)
Supplement: Supplementary file 1 [file jcm-13-06793-s001.zip › jcm-3253457-supplementary.pdf]

Supplementary Material

Supplementary Figure S1

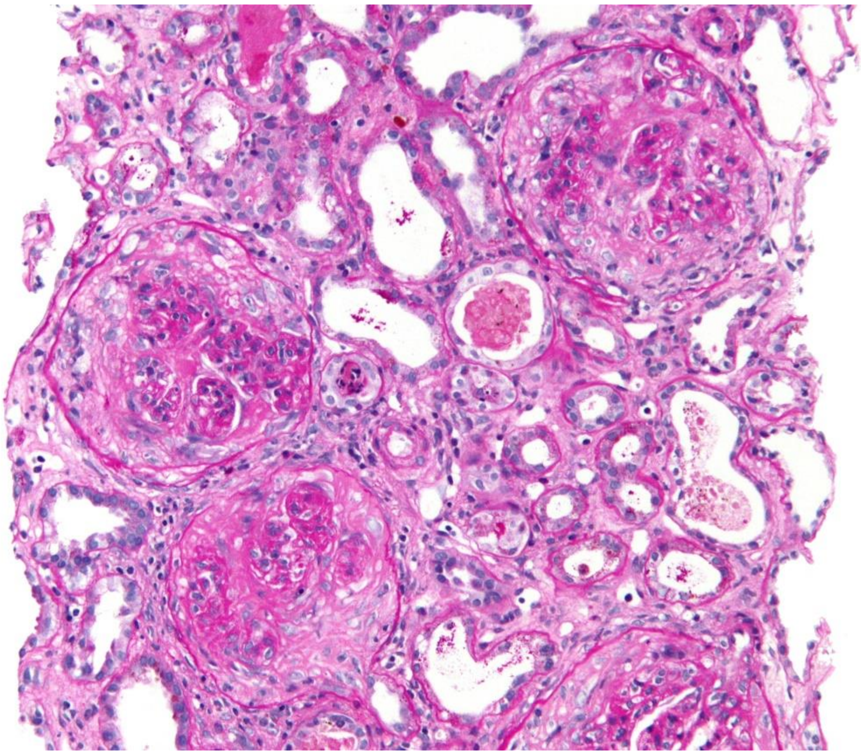

Necrotizing glomerulonephritis with extracapilary proliferation in a patient with ANCA Associted vasculitis

Supplementary Table S1

Levels of Evidence		Grades of recommendation	
**Level 1**	Systematic reviews of randomised controlled trials. Individual RCTs with narrow confidence interval and all or none cases when the effects of the intervention are not clinically disputable.	**Evidence A**	Strong recommendation. Based on **Level 1** studies. The quality of the evidence available is the highest possible. The recommendation is expected to be followed and used as a quality indicator.

**Level 2**	Systematic reviews of cohort studies, individual cohort studies and low-quality RCTs Study of results.	**Evidence B**	Less-strong recommendation. Based on **Level 2 studies** or extrapolations of Level 1 studies. The quality of the evidence available is high or moderate, so along with other considerations this recommendation should be followed. It is expected to be followed by most clinics.
**Level 3**	Systematic or individual reviews of case-control studies.	**Evidence C**	Weak recommendation. Based on **Level 3 or 4 studies or extrapolations of Level 2 studies.** The recommendation should be followed.
**Level 4**	Case series (or poor-quality cohort and case-control studies).		
**Level 5**	Expert opinions.	**Evidence D**	Opinion. The quality of the evidence available is low or very low **(Level 5).** It is a recommendation based on expert opinions.

Evidence was graded according to the *Levels of Evidence and Grades of Recommendation* by the Centre for Evidence-Based Medicine (Oxford University)
